# The Intracellular Cleavage Product of the NG2 Proteoglycan Modulates Translation and Cell-Cycle Kinetics via Effects on mTORC1/FMRP Signaling

**DOI:** 10.3389/fncel.2018.00231

**Published:** 2018-08-07

**Authors:** Tanmoyita Nayak, Jacqueline Trotter, Dominik Sakry

**Affiliations:** ^1^Department of Biology, Molecular Cell Biology, Institute of Developmental Biology and Neurobiology, Johannes Gutenberg University Mainz, Mainz, Germany; ^2^Department of Molecular Neurobiology, Max Planck Institute for Experimental Medicine, Göttingen, Germany

**Keywords:** NG2, ICD, γ-secretase, OPC, FMRP, mTOR, S6K1, eEF2

## Abstract

The NG2 proteoglycan is expressed by oligodendrocyte precursor cells (OPCs) and is abundantly expressed by tumors such as melanoma and glioblastoma. Functions of NG2 include an influence on proliferation, migration and neuromodulation. Similar to other type-1 membrane proteins, NG2 undergoes proteolysis, generating a large ectodomain, a C-terminal fragment (CTF) and an intracellular domain (ICD) via sequential action of α- and γ-secretases which is enhanced by neuronal activity. Functional roles of NG2 have so far been shown for the full-length protein, the released ectodomain and CTF, but not for the ICD. In this study, we characterized the role of the NG2 ICD in OPC and Human Embryonic Kidney (HEK) cells. Overexpressed ICD is predominantly localized in the cell cytosol, including the distal processes of OPCs. Nuclear localisation of a fraction of the ICD is dependent on Nuclear Localisation Signals. Immunoprecipitation and Mass Spectrometry followed by functional analysis indicated that the NG2 ICD modulates mRNA translation and cell-cycle kinetics. In OPCs and HEK cells, ICD overexpression results in an mTORC1-dependent upregulation of translation, as well as a shift of the cell population toward S-phase. NG2 ICD increases the active (phosphorylated) form of mTOR and modulates downstream signaling cascades, including increased phosphorylation of p70S6K1 and increased expression of eEF2. Strikingly, levels of FMRP, an RNA-binding protein that is regulated by mTOR/p70S6K1/eEF2 were decreased. In neurons, FMRP acts as a translational repressor under activity-dependent control and is mutated in Fragile X Syndrome (FXS). Knock-down of endogenous NG2 in primary OPC reduced translation and mTOR/p70S6K1 phosphorylation in Oli-*neu*. Here, we identify the NG2 ICD as a regulator of translation in OPCs via modulation of the well-established mTORC1 pathway. We show that FXS-related FMRP signaling is not exclusive to neurons but plays a role in OPCs. This provides a signal cascade in OPC which can be influenced by the neuronal network, since the NG2 ICD has been shown to be generated by constitutive as well as activity-dependent cleavage. Our results also elucidate a possible role of NG2 in tumors exhibiting enhanced rates of translation and rapid cell cycle kinetics.

## Introduction

NG2 (*CSPG4*) is a type-1 membrane protein (300 kD MW), belonging to the chondroitin sulfate proteoglycan protein family with a large extracellular region (290 kD) and a very short intracellular domain (8.5 kD). It was first discovered in the rat nervous system (Stallcup, 1977) and has reported homologs in mouse (Niehaus et al., 1999; Schneider et al., 2001; Stegmüller et al., 2002), human (Pluschke et al., 1996), and Drosophila (Estrada et al., 2007; Schnorrer et al., 2007).

In the nervous system, oligodendrocyte precursor cells (OPC) express the NG2 protein, while neurons (Karram et al., 2008; Clarke et al., 2012), astrocytes (Zhu et al., 2008; Huang et al., 2014) and resident microglia (Moransard et al., 2011) do not. Additionally, a subpopulation of pericytes, cells from the vascular system, also express the NG2 protein (Ozerdem et al., 2002; You et al., 2014; Attwell et al., 2016). NG2 is expressed by many tumor types, including glioblastoma multiform (GBM) and melanoma (Chekenya et al., 2008; Prestegarden et al., 2010; Al-Mayhani et al., 2011), and is thus considered a potential therapeutic target in cancer.

OPCs are a self-renewing, and proliferating cell population found at all stages of development in gray and white matter. In the adult mammalian brain, around 5% of total neural cells comprise the OPC cell population (Dawson et al., 2003). During differentiation of OPCs into myelinating mature oligodendrocytes, NG2 expression is down-regulated (Nishiyama et al., 2009; De Biase et al., 2010; Kukley et al., 2010). NG2+ OPCs are unique as they are the only glial cell population receiving direct synaptic input from neurons (Bergles et al., 2000; Jabs et al., 2005; Kukley et al., 2008; Mangin and Gallo, 2011; Sakry et al., 2011).

**Figure 1:**
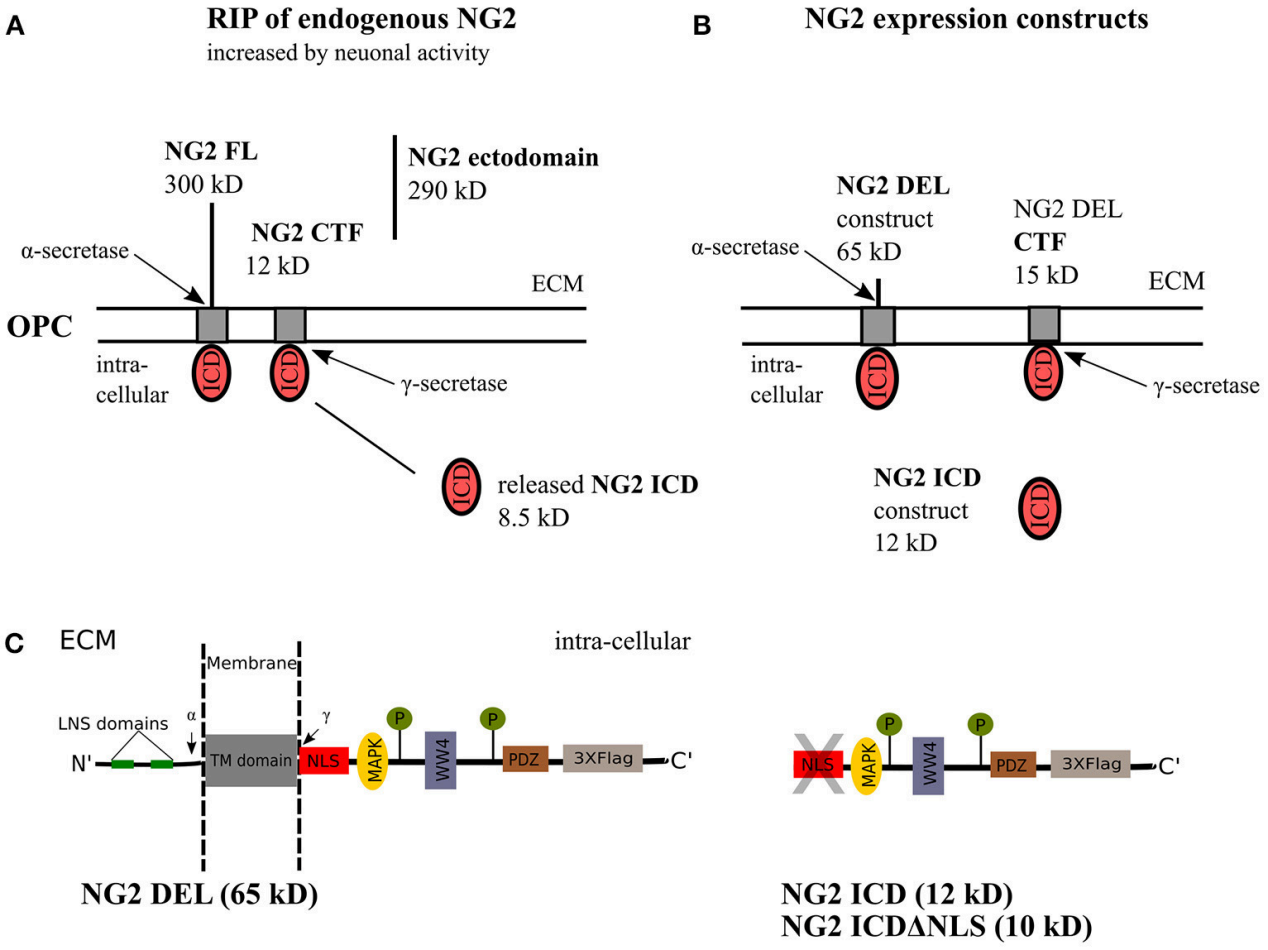
NG2 cleavage and expression constructs. **(A)** In OPCs, NG2 full-length (FL) protein undergoes regulated intramembrane proteolysis (RIP) leading to the release of an ectodomain into the extracellular matrix (ECM) and generation of a c-terminal fragment (CTF) by α-secretase activity. γ-secretase activity on the NG2 CTF releases the NG2 intracellular domain (ICD) into the cytoplasm. This NG2 cleavage happens constitutively in OPCs and can be increased by neuronal activity over Neuron-OPC synaptic innervations (Sakry et al., 2014). **(B)** The NG2 DEL and NG2 ICD expression construct used in this study. NG2 DEL (65 kD) mimics full-length NG2 (300 kD) but comprises one large deletion within the ectodomain, it can be processed by α- and γ-secretases like the FL protein as outlined in **(A)**. The NG2 ICD expression construct mimics released NG2 ICD by γ-secretase. **(C)** Sequence overview of the NG2 DEL, ICD and ICDΔNLS expression construct. The NLS, WW4 and MAPK binding motif have been predicted by an ELM database (elm.eu.org) analysis. The highlighted LNS domains, the two phosphorylation sites, and the PDZ binding motif have distinct cellular functions as described in the introduction.

Regulated intramembrane proteolysis (RIP) of type-1 membrane proteins involves sequential cleavage by α- and γ-secretases. The action of endogenous α-secretase on the full-length (FL) protein leads to release of the ectodomain into the extracellular matrix, termed as shedding. Subsequent cleavage of the remaining C- terminal fragment (CTF) by the γ-secretase releases the intracellular region into the cytoplasm, the released peptide is a functional unit and therefore termed intracellular domain (ICD), also referred to as the released ICD in the following text (Brown et al., 2000; Lal and Caplan, 2011; Saftig and Lichtenthaler, 2015). Notch was the first-reported, well-characterized membrane-protein undergoing RIP (Levitan and Greenwald, 1995; Blaumueller et al., 1997; De Strooper et al., 1999) and the released Notch ICD exhibits defined cellular functions, acting as a transcription factor (Bailey and Posakony, 1995; Baker and Zitron, 1995). Although release of the NG2 ectodomain had been previously documented (Nishiyama et al., 1995; Deepa et al., 2006), our published work demonstrated that NG2 undergoes RIP, generating a CTF and ICD in OPCs (Sakry et al., 2014, 2015; Sakry and Trotter, 2016). Recently, RIP has been shown for the neuronal CNS proteins neuroligin-1 and N-cadherin (Malinverno et al., 2010; Suzuki et al., 2012). Interestingly, this process is enhanced by neuronal activity, and similarly, we demonstrated that RIP of NG2 was also enhanced by activity (Sakry et al., 2014; Figure 1A). Thus, the cleavage and signaling pathways of these membrane proteins can be potentially modulated by the neuronal network.

The intracellular region of NG2 is known to bind to scaffold proteins such as Mupp1, Syntenin and GRIP1(Barritt et al., 2000; Stegmuller et al., 2003; Chatterjee et al., 2008), and protects cell from oxidative stress by binding to OMI/HtrA2 (Maus et al., 2015). It has also been associated with directed migration of OPCs (Binamé et al., 2013) and has a regulatory influence on the expression of the neuromodulator enzyme PTGDS in OPCs (Sakry et al., 2015).

In this study, we focused on basic cellular functions and signaling pathways which are specifically altered by the released NG2 ICD in OPCs. This is especially important because it would directly demonstrate the NG2 ICD is a functional domain. Additionally, NG2 cleavage-dependent pathways in OPCs are potentially regulated by the neuronal network. The NG2 ICD signaling cascade that we describe may be pertinent for other NG2 expressing cell-types, especially tumors.

## Materials and methods

### Cell culture

The OPC cell-line, Oli-*neu*, was used as described in Jung et al. (1995). Cells were cultured at 37°C, with 5% CO_2_ and expanded in Sato medium containing 1% horse serum [DMEM, with 0.2% (w/v) sodium bicarbonate, 0.01 mg/ml insulin, 0.01 mg/ml transferrin, 220 nm sodium selenite, 100 μm putrescine, 500 nm triiodothyronine, 520 nm thyroxine, and 200 nm progesterone]. HEK 293 (HEK, Invitrogen) cells were cultivated at 37°C in DMEM (Sigma) with 1% pyruvate and 10% FCS with 8% CO_2_. For primary OPC (pOPC), single cell suspensions were obtained from total brains of the Postnatal day (P) P6-9 of C57Bl/6N mice using the NTDK-P Kit (Miltenyi Biotec). Magnetic isolation (MACS) was performed as described previously (Diers-Fenger et al., 2001). 300,000 cells were plated in each well of a PLL-coated dish (6-well format) and cultured in OPC proliferation medium. OPC proliferation medium consists of NeuroMACS medium (Miltenyi) supplemented with 1:50 of NeuroBrew Miltenyi, 1:100 of L-Glutamine, 1:100 Pen-strep and 1:1,000 of stock conc. of each of Forskolin (4.2 mg/ml), CNTF (10 μg/ml), PDGF (10 μg/ml) and NT-3 (1 μg/ml). pOPCs were maintained at 37°C with 8%CO_2_. All animal experiments were carried out in strict accordance with protocols approved by local Animal Care and Use Committee of Johannes Gutenberg University of Mainz. Mice were sacrificed by decapitation to remove the brain.

### Expression vectors and transfection

NG2 expression vectors were derived from pRK5 constructs used in Sakry et al. (2015) by PCR amplification. NG2 DEL expresses a short recombinant version of full-length NG2 (consisting the signal sequence, one-fourth of the extracellular portion including two laminin G-type motifs, a transmembrane domain, and intracellular domain). Two other constructs are expressing only the intracellular domain (NG2 ICD, AA 2251-2327, UniProt: Q8VHY0) and without the predicted Nuclear Localization Signal (NLS) of ICD (NG2 ICDΔNLS, AA 2259–2327). All cDNAs were cloned into the p3X-Flag CMV14 expression vector (c-terminal 3X flag-tag) and used for transfection. Additionally, the H2B-GFP plasmid (gift of Dr. Vijay Tiwari) was co-transfected along with NG2 ICD-Flag expression vector for immunocytochemistry study.

HEK and Oli-*neu* cells were plated 1 day before transfection and transfected using either PEI or Fugene HD reagent (Promega) at a ratio of 1:3 (2 μg DNA: 6 μl Fugene for the 3 cm dish). 48 h after transfection, cells were harvested and processed for analysis.

Primary OPCs were transfected after 1 day *in vitro* (DIV 1) using Lipofectamine RNAiMAX reagent (Thermo Fisher) according to the protocol. 120 pmol siRNA (final concentration) was used per well (6-well format), and the medium was changed 5–6 h after transfection. Cells were processed for analysis at DIV 2.

### Cell lysates, SDS PAGE, and western blotting

Cells were washed with PBS and scraped with a rubber policeman into lysis buffer (PBS, 1% TX-100, 1X protease inhibitor (PI) cocktail from Roche) from the culture plate on ice. After incubation for 20 min on the rotor at 4°C, cells were spun down by centrifugation at 1,000 g, 10 min, 4°C. Supernatants were defined as postnuclear (PN) cell-lysates (lysates). The same volume of lysis buffer was used per sample, and all samples were diluted with 4x SDS or LDS (Invitrogen) sample buffer, heated to 80°C for 10 min and resolved on 4–12% NuPage Bis-Tris gradient gel in combination with MES or MOPS running buffer (Invitrogen). Western blotting (WB) was done with NuPage Blot system utilizing a PVDF membrane (Millipore). The latter was blocked for 30 min in PBS containing 0.1% Tween 20 (PBST) and 4% nonfat milk or 4% BSA.

Blocked membranes were incubated with primary antibodies (AB) overnight at 4°C in blocking solution, followed by three washes (PBST). Subsequently, they were incubated with 1:10,000 HRP-conjugated secondary AB (Dianova) in blocking solution for 1 h and washed for three times again. Signal detection was carried out using enhanced chemiluminescence (ECL) assay solution (Millipore) and hyperfilms (GE). ImageJ 1.46 (NIH) was used for signal quantification, and all protein levels were normalized against GAPDH from the same sample. In some experiments, for checking total loaded protein level, membranes were stained with Ponceau S solution for 5 min on a shaker and later rinsed with deionized water three times for 5 min each.

### Sub-cellular fractionation assay

For subcellular fractionation, cells were plated 1 day before transfection and transfected with NG2 ICD or ICDΔNLS- Flag plasmids. After 48 h, cells were lysed with cytosolic lysis buffer (1X PBS+ 1% NP-40, 1X PI) on ice for 30 min and centrifuged at 2,000 g for 10 min. Supernatants which were enriched with cytosolic fraction were collected. The pelleted nuclei were further digested with nuclear lysis buffer [20 mM Tris-HCl (pH 7.4), 150 mM NaCl, 10 mM MgCl2, 1% TX-100, 2.5 mM Beta-glycerophosphate, 1 mM NaF, 1 mM DTT, 2 mM EDTA, 10% glycerol 10U of Benzonase, 1X Protease inhibitor cocktail]. for 1 h on a rotor at 4°C. Samples were centrifuged at 7,000 g for 10 min, and the supernatant was collected which comprises nuclear proteins.

### Immunoprecipitation (IP) and mass spectrometry (MS)

Cells were plated in 100 mm dishes, and at ~80% confluency were transiently transfected with 8 μg plasmid DNA of ICD and BAP-flag tagged constructs. After 48 h, cells were washed, lysed (1x PBS+ 0.5% TX-100+ 1x protease inhibitor), scraped off and centrifuged (3000 g). Prior to IP, the supernatant was precleared at 12,000 g for 10 min and incubated with anti-flag M2 magnetic beads (Sigma) for 2 h on a rotor at 4°C. The beads were collected and washed three times (with PBS+0.3% TX-100) and heated at 85°C for 10 min with 2X LDL sample buffer. Later, the IPed samples were used for various functional assays (coomassie staining, Mass Spec, CoIP). Mass-spectrometry based analysis of the IPed samples was done by chemical labeling method (DML labeling) in both forward and reverse way (IMB, Mainz). Data was analyzed by Maxquant software where the potential contaminants were removed, and the threshold was set to a minimum of 2X enrichment.

### Primary antibodies

The following antibodies are used for WB, ICC or FC: 1:200 (WB) of rabbit anti-NG2 cyto (Stegmüller et al., 2002), 1:1,000 (WB) of mouse anti-Flag (Sigma, F1804); 1: 200 (FC) of anti-Flag-FITC (Sigma, F4049); 1:1,000 (WB) of rabbit anti-FMRP (Sigma, F4055); 1:1,000 (WB) of rabbit anti-eEEF2 antibody (Abcam, ab40812); 1:1,000 (WB) of rabbit anti-eIF4B (CST, 3592T); 1:1,000 (WB) of rabbit anti-phospho (Ser422) eIF4B (CST, 3591S); 1:200 (ICC) of rabbit anti-PCNA (NEB, 13110); 1:1,000 (WB) of mouse anti-Puromycin (Millipore, MABBE343); 1:5,000 (WB) of rabbit anti-GAPDH (Biomol, A-300-641A); 1:400 (WB) of mouse anti-Cyclin E (SCBT, E-4), 1:1,000 (WB) of rabbit anti-p70S6K1 (CST, 9202). For studying mTOR and p70S6K1 phosphorylation, substrate sampler kit (CST, 9862T) was used and the antibody dilutions were made for WB according to kit suggestions.

### Immunocytochemistry

Oli-*neu* cells were cultured on poly-L-Lysine coated coverslips, washed with PBS and fixed with 4% PFA for 15 min at RT, washed and permeabilized for 5 min with PBS containing 0.3% TX-100. Blocking was with PBS+10%HS for 30 min at RT. Primary AB incubation was done overnight at 4°C, and secondary antibodies were applied for 30 min at RT. Coverslips were mounted in Moviol. For PCNA staining, cells were washed (1X PBS) and incubated with PBS+ 0.3% Triton-X on ice for 5 min and immediately fixed with acetone: methanol (1:1) solution for 10 min at −20°C. Later, cells were blocked with PBS containing 3% goat serum for 1 h at RT. Primary and secondary AB incubation was done as described above. DAPI (0.1 μg/ml) was added along with secondary antibodies. The following secondary AB were used: goat or donkey anti mouse and anti-rabbit coupled with Alexa488, 546 or 647 (Invitrogen).

Images were taken with a Leica DM6000 fluorescent microscope or an SP5 laser scanning confocal microscope (IMB, Mainz). Image processing was done with Leica LAS AF and ImageJ (NIH), specifically the ImageJ3DViewer. Nuclear morphology of immunostained cells were also analyzed for two indices; area and roundness with ImageJ.

### SUnSET assay

HEK293 and Oli-*neu* cells were transiently transfected with either NG2 ICD or empty flag control vector. After 48 h, cells were incubated with puromycin (10 μg/mL) for 15 min in the culture medium and lysed afterwards for WB as described above. Anisomycin, a translation blocker, was used as a negative control (40 μM final concentration) to confer puromycin incorporation only in active ongoing nascent polypeptide chains. The same protocol was repeated with siCNT, and siNG2 transfected primary OPCs and puromycin incubation was carried out 24 h post-transfection.

In an additional and independent experiment, Oli-*neu* cells were treated with a mTORC1 inhibitor, Temsirolimus (ab141999). Cells, overexpressing control or ICD, were treated with Temsirolimus (TM) at a final concentration of 12 μM for 24 h and then treated with Puromycin as previously described. Cells were subsequently lysed (PBS + 0.5% TX-100 + 1X PI), and lysates were used for WB and signals developed after incubation with the anti-puromycin antibody. Before Western-Blotting, total loading of puromycin-treated protein samples was checked by Ponceau S staining.

### Facs

HEK293 cells were transfected with NG2 ICD or BAP-flag (as a positive control) and pelleted in a falcon after 48 h by centrifugation (800 rpm, 10 min, 4°C). Cells were fixed with pre-chilled 80% ethanol in a dropwise manner and incubated for 10 min on ice. After permeabilization (PBS, 0.3% TX-100, 1X PI) and blocking with PBS+10% HS for 1 h, cells were incubated with the primary anti-Flag-FITC conjugated antibody (in permeabilization buffer) for 2 h at 4°C. For DNA content analysis, cells were treated with RNase (10 μg/mL) and stained with propidium iodide (50 μg/mL) for 30 min at dark. Cell cycle distribution was checked only for Flag+ cell population, and data were analyzed by FlowJo software (IMB, Mainz).

### Statistics

Each experiment was repeated independently at least three or more times. The numerical data of multiple experiments is expressed as mean ± standard error of the mean (SEM). Statistical analysis was done using Excel and Graph Pad Prism. For significance analysis, normal distribution was tested by the Shapiro-Wilk Normality test. For parametric distributed datasets two tailed *t-*test was applied: In the case of non-parametric distribution, Mann-Whitney or Wilcoxon Signed rank test was used. Significance was classified as follows: ^*^p ≤ 0.05; ^**^p < 0.01; ^***^p < 0.001; n.s. *p* > 0.05.

## Results

### NG2 expression constructs

As a tool for analyzing the cellular localization and functions of NG2 ICD, the following expression constructs were used (for details see the Methods section). NG2 DEL (65 kD) mimics a shortened version of the full-length NG2 protein, NG2 ICD (12 kD) encodes the intracellular domain of NG2 and ICDΔNLS (10 kD) encodes the ICD lacking the first nine amino acids which consist the predicted Nuclear Localization Signal(s). All these constructs were c-terminally tagged with three flag epitopes (p3X-Flag-CMV-14, Sigma) and are shown in detail in Figures 1B,C.

### NG2 ICD localizes predominantly to the cytosol and distal processes with limited nuclear expression

**Figure 2:**
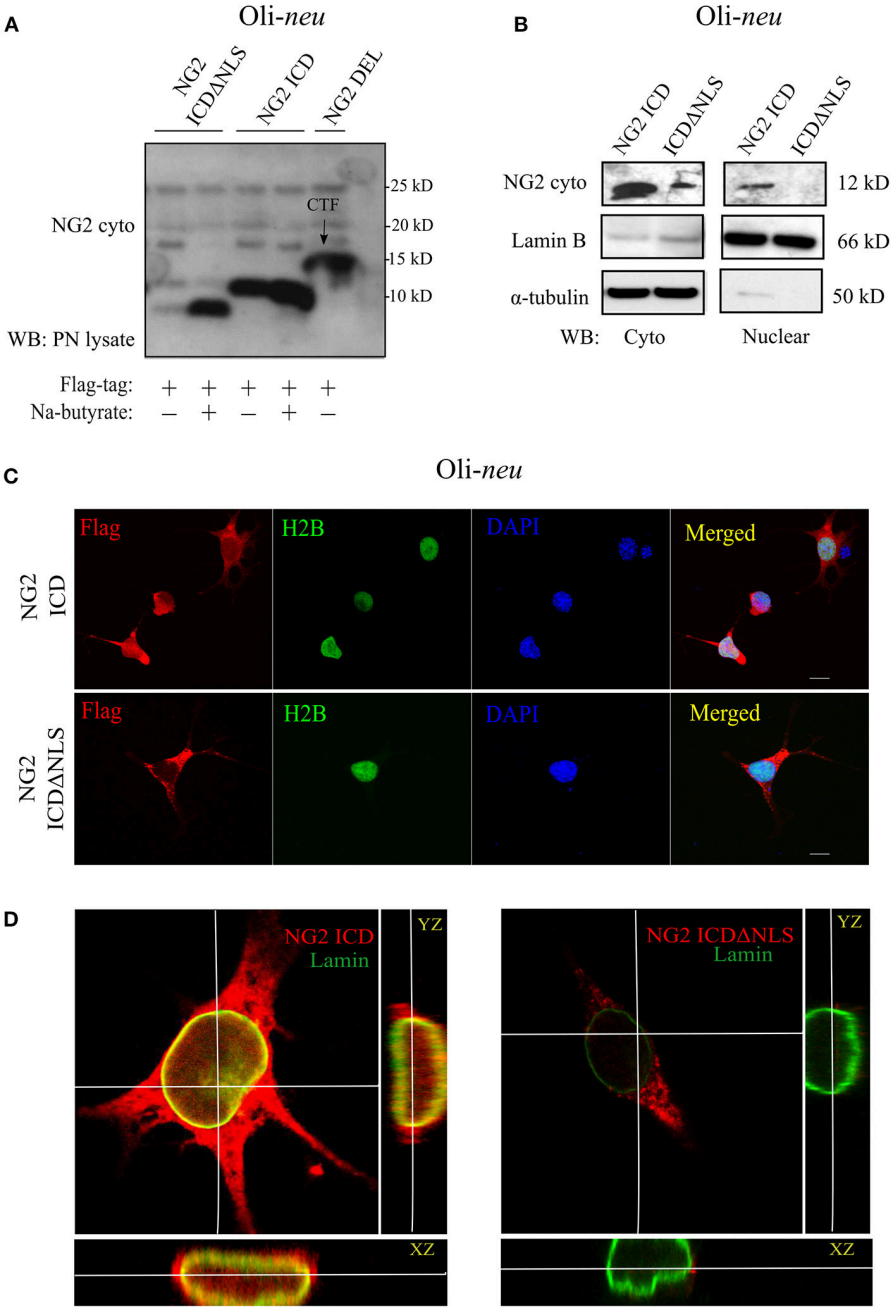
Cellular localization of NG2 ICD in cultured OPCs. **(A)** Western blot analysis of post-nuclear (PN) cell-lysates of the OPC cell-line, Oli-*neu*. Different NG2 expression-constructs have been transiently transfected for 48 h before cell-lysis. Flag-tagged constructs show their size-based differences, NG2 ICD runs at 11 kD, and NG2 ICDΔNLS (NLS was deleted) runs just below ICD at 10 kD. NG2 DEL (65 kD) strongly shows processed membrane-bound CTF (15 kD) at the shown lower molecular masses. Na-butyrate was used only in this experiment to enhance the expression of ICD constructs. **(B)** Sub-cellular fractionation assay was done from Oli-*neu* after overexpressing ICD or ICDΔNLS for 48 h. Lamin B and α-tubulin were used as nuclear and cytosolic markers respectively. **(C)** Confocal laser scanning microscope images (z-stack, max. intensity projection) of Oli-*neu* expressing NG2 ICD or ICDΔNLS (flag; red) fixed after 48 h of transfection. Cells were immunostained with anti-flag antibody. NG2 ICD shows a distinguishable homogeneous staining pattern including nucleus where in case of ICDΔNLS, expression remained cytosolic. H2B (green) and DAPI (blue) were used as nuclear markers. **(D)** Three-dimensional orthogonal view of a z-stack of an Oli-*neu* showed the presence of NG2 ICD (red) within the nucleus, whereas ICDΔNLS expression was limited to the cytosol and peri-nuclear area. Lamin B (green) was used as a nuclear envelope marker. (Scale bar = 10 μm).

First, we checked the expression of the different NG2 constructs and their size-difference pattern in a gradient gel by western blot analysis (WB). The HEK 293 cell-line (HEK), as well as the OPC cell line, Oli-*neu* and primary OPCs (pOPC) from total brain (mouse), were routinely used for transfection. The small endogenous NG2 ICD has an MW of 8.5 KD and thus, like other gamma-secretase products, was unstable and hard to detect in WB with a specific antibody recognizing the intracellular part of NG2 (NG2-cyto). To initially validate the expression of ICD constructs, we used sodium butyrate to enhance the expression levels. However, sodium butyrate was not used in any other experiments. Expression of ICDΔNLS only lead to sufficient protein levels in WB if sodium butyrate was added, while NG2 ICD levels were high even in the absence of butyrate. NG2 CTF (15 kD) derived from NG2 Del expression construct was the predominant, membrane-bound cleavage product recognized by the NG2 cyto antibody (Figure 2A). The localization of NG2 ICD and function of the predicted NLS was analyzed by performing subcellular fractionation of ICD over-expressing Oli-*neu* cells. NG2 ICD was detected in the nuclear fraction (Nuclear) as well as in the cytosolic fraction (Cyto) while expression of ICDΔNLS was restricted to the cytosolic fraction (Figure 2B). These observations were confirmed when Oli-*neu* cells transiently expressing the NG2 ICD and ICDΔNLS were immunostained and analyzed by confocal microscopy (Figures 2C,D). However, the cytosolic levels of ICD were always higher than the levels in the nucleus.

### Identification of the NG2 ICD interactome

**Figure 3:**
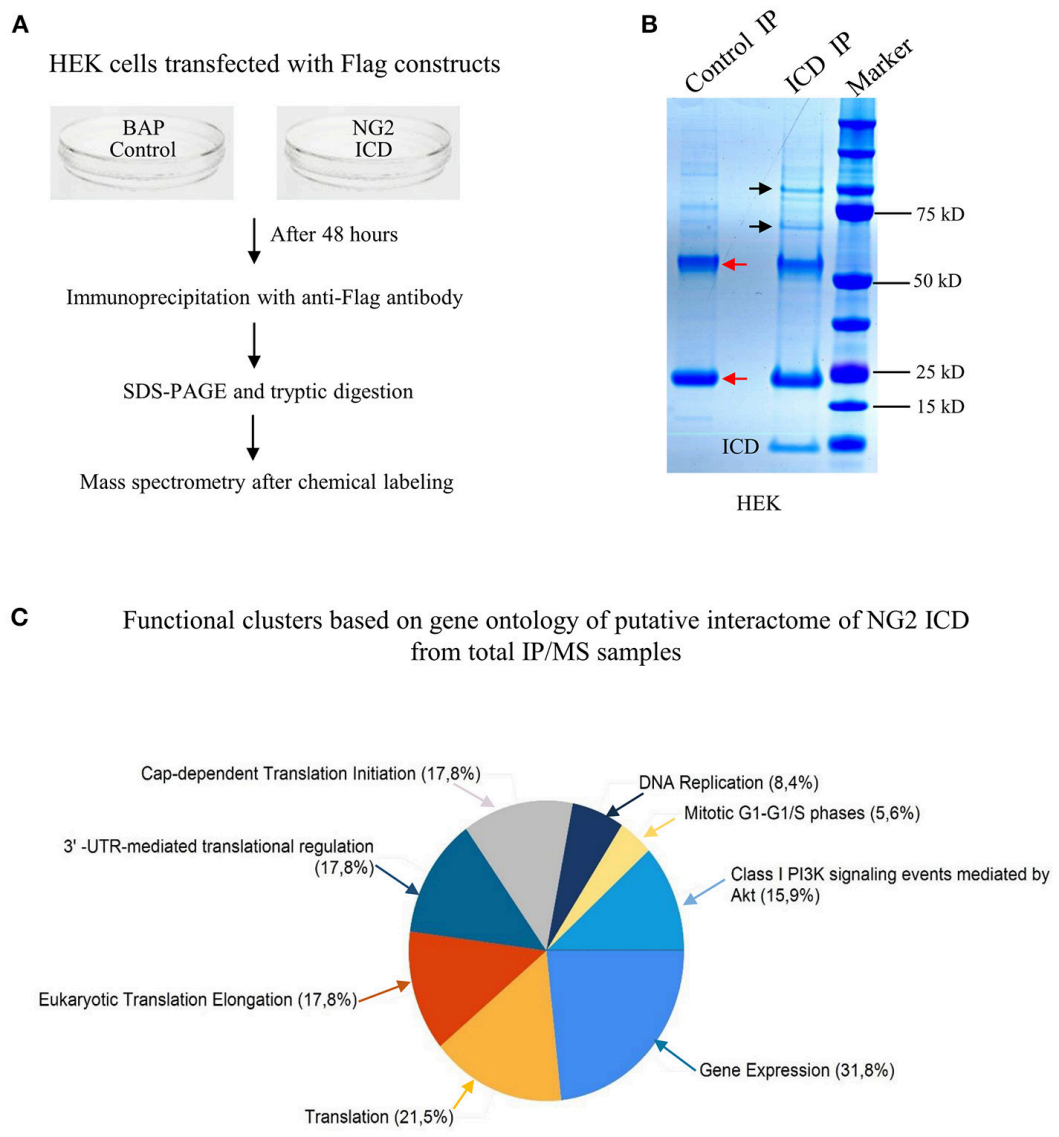
Screening for the NG2 ICD interactome by IP-MS demonstrated enrichment of translation cluster. **(A)** Schematic of experimental conditions, immunoprecipitation and Mass spectrometry (MS) to isolate proteins that potentially interact with released NG2 ICD in HEK cells. Bacterial Alkaline Phosphatase (BAP) expression construct was used as control (control). **(B)** Representative Coomassie-stained gradient gel of NG2 ICD (ICD IP) and BAP-overexpressing samples (Control IP) after IP. Unique bands present in ICD-IP lane are indicated by black arrows, and red arrows indicate heavy and light chain (55 and 25 kD respectively) from anti-flag IgG used for IP. The unique bands were excised from gel between 70 and 100 kD and subjected to MS-based analysis. **(C)** MS analysis of whole IPed samples was carried out in two independent experiments, each time with additional reversed peptide-labeling. The putative IP-MS candidates were analyzed by using FUNRICH analysis tool. Altered biological functional annotation based on gene ontology represented translation, ribosomal biogenesis, and DNA replication to be significantly enriched.

Since the major fraction of the ICD was present in the cell cytosol and the NG2 ICD has several predicted protein binding motifs, we characterized the interactome of the NG2 ICD. HEK cells were transiently transfected with NG2 ICD or BAP (Flag-tagged control protein) and immunoprecipitation was carried out using magnetic anti-Flag antibody conjugated beads (Scheme shown in Figure 3A). Proteins that co-precipitated with NG2 ICD were identified by mass spectrometry (MS) from total IP samples run on gradient SDS-PAGE. Red arrows in Figure 3B indicate heavy (55 kD) and light chain (25 kD) of the Flag IgG used for IP. The NG2 ICD (around 12 kD) is also shown on the gel image, while BAP (control) protein (around 51 kD) overlaps with the IgG heavy chain and is indistinguishable from the latter on the Coomassie gel. Additionally, in an independent experiment, observed unique bands in ICD IP lane from the Coomassie-stained gradient gel were excised between 70 and 100 kD and sent for mass spectrometry-based band identification analysis (indicated by black arrows in Figure 3B). Band identification revealed that peptides corresponding to *two* translation factors eEF2 (100 kD) and eIF4B (70 kD) were highly represented in these excised bands (Table 1). Strikingly, KEGG biological pathway analysis based on Gene ontology from whole NG2 ICD-IPs also suggested an enrichment of DNA replication, translation regulators, PI3K-Akt-mTOR signaling pathways and cell-cycle stages (Figure 3C). Additionally, a functional annotation clustering analysis of the putative interaction partners of NG2 ICD was performed with FUNRICH proteomic analysis tool. This analysis also confirmed that protein clusters involved in translation regulation, cell-cell adhesion RNA metabolism, and DNA replication were highly represented in the identified annotated peptide list (Table 2).

**Table 1:** Unique band identification of IPed samples by MS revealed enrichment of two translation factors in ICD IP lane.

**Gene name**	**Protein name**	**UniProt ID**	**Molecular weight**	**Unique peptides**	**Sequence coverage**
EEF2	Eukaryotic elongation factor 2	P13639	95.33	24	32.4
EIF4B	Eukaryotic initiation factor 4B	P23588	66.12	15	28.8

**Table 2:** Functional annotation clusters of putative interactome of cleaved NG2 ICD by FUNRICH analysis software from whole IP/MS data.

	**count(% total)**	***p*-value**
**GO-MOLECULAR FUNCTION**		
RNA binding	75 (58.1)	1.1E-41
Nucleotide binding	46 (35.7)	3.0E-11
Cadherin binding involved in cell-cell adhesion	14 (10.9)	7.0E-6
**GO-BIOLOGICAL PATHWAY**		
Ribosome	20 (15.05)	5.25E-15
Proteasome	5 (3.87)	0.002
PI3K-Akt Signaling pathway	4 (3.1)	0.807
**GO-CELLULAR COMPARTMENT**		
Ribosomal complex	22 (17.05)	3.04E-20
Spliceosomal complex	7 (5.42)	5.38E-5
ER-Chaperone complex	4 (3.1)	5.67E-5

### NG2 ICD increases translation rate in cultured OPCs in an mTORC1-dependent manner

**Figure 4:**
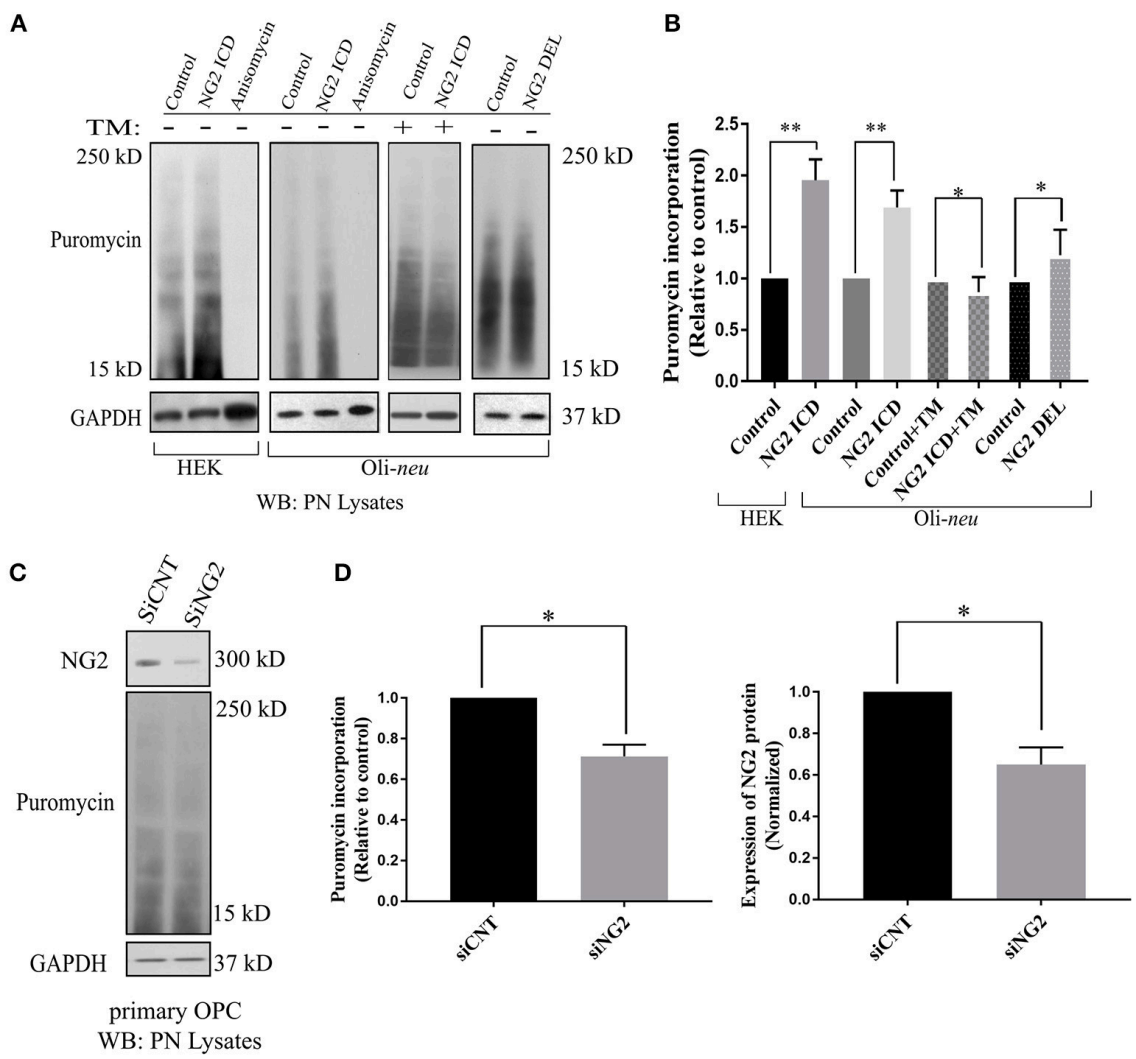
Released NG2 ICD stimulates *in-vitro* translation in a mTORC1-dependent manner. **(A)** HEK (*n* = 5) and Oli-*neu* (*n* = 4) cells were treated with 10 μM puromycin after 48 h of transient transfection with different NG2 expression constructs (DEL and ICD) or empty expression vector (control). Puromycin incorporates into newly synthesized proteins during translation. Western blot analysis of post-nuclear (PN) cell-lysates after puromycin treatment was done with a specific antibody against puromycin, the translation blocker Anisomycin was used as a negative control. Additionally, a selective mTORC1 complex inhibitor, Temsirolimus (TM), was used (10 μM) to investigate mTORC1 specific contribution to total translation rate between control (+TM) and ICD (+TM) (*n* = 3). **(B)** Densitometric analysis was done for puromycin (from 15 to 250 kD) normalized to GAPDH. NG2 ICD showed an increase of puromycin incorporation (translation rate) by 70% in Oli-*neu* and by 90% in HEK cells compared to control. When treated with a mTORC1 inhibitor (TM), the ICD-mediated effect on translational is reduced slightly below control levels (control+TM vs. ICD+TM), indicating a mTORC1-dependent increase and a mTORC1-independent decrease of translation by NG2 ICD overexpression. NG2 DEL mimics full-length NG2, and it predominantly expresses membrane-bound NG2 CTF but not cleaved ICD (Figures 1B, 2A). NG2 DEL was used as an additional control to confer cleaved ICD-specificity in stimulating translation, and it shows only ~22% increase in translation rate compared to control (*n* = 4). **(C)** Full-length NG2 knockdown (siRNA transfection) was performed in cultured primary OPCs (pOPCs) to check the effect on translation. **(D)** NG2 knockdown (siNG2) in pOPCs revealed a knockdown efficiency of around 40% compared to control (siCNT), translation (puromycin level) was reduced by ~30% by NG2 knock-down. [Data represents mean ±SEM. Statistical analysis was done by two-tailed paired *t*-test after checking the data is normally distributed by Shapiro-Wilk normality test by PRISM (GraphPad)]. Significance was classified as defined in the Materials and Methods part.

The GO-based analysis strongly suggested an influence of the NG2 ICD on translation. To test this directly, we pulse-labeled Oli-*neu* and HEK cells with puromycin followed by immunoblotting with an anti-puromycin antibody, a technique known as SUnSET assay (Schmidt et al., 2009). Low concentrations of puromycin are incorporated into nascent polypeptide chains labeling the newly synthesized proteome. Increased puromycin incorporation reflects a higher translation rate (Figure 4A). The translation blocker Anisomycin was used as negative control. In cells overexpressing ICD, puromycin incorporation was increased by ~90% in HEK and ~70% in Oli-*neu* cells compared to controls, indicating an increased translation rate (Figure 4B). To validate that the observed increased translation rate is specific for cleaved NG2 ICD, NG2 DEL was used. NG2 DEL mimics membrane-bound NG2 and is cleaved in the cell yielding small amounts of ICD. Expression of NG2 DEL in Oli-*neu* yielded a smaller increase in the puromycin signal compared to control (~22%). Specific inhibition of mTORC1 by temsirolimus (TM) completely blocked the NG2 ICD dependent increase of translation in Oli-*neu* (ICD + TM) compared to the control (TM) (Figure 4B) and reduced the translation rate to slightly below control levels. Knock-down of endogenous NG2 (siNG2) in primary OPC by siRNA reduced NG2 levels by 40% and total translation was also reduced by around 30% in pOPC transfected with siNG2 compared to control (Figures 4C,D).

### NG2 ICD increases cell population in S-phase

The proteomics data showed that DNA replication was one of the top altered processes in cells overexpressing ICD. Furthermore, exaggerated protein synthesis often leads to cell proliferation (Bilanges and Stokoe, 2007). We thus investigated whether cleaved NG2 ICD plays a role in cell-cycle regulation.

HEK cells were transfected with NG2 ICD or BAP-Flag (Flag positive control). After 24 h, the cells were fixed and stained with anti-Flag-FITC conjugated antibody after permeabilization and analyzed by FACS after staining with PI.

**Figure 5:**
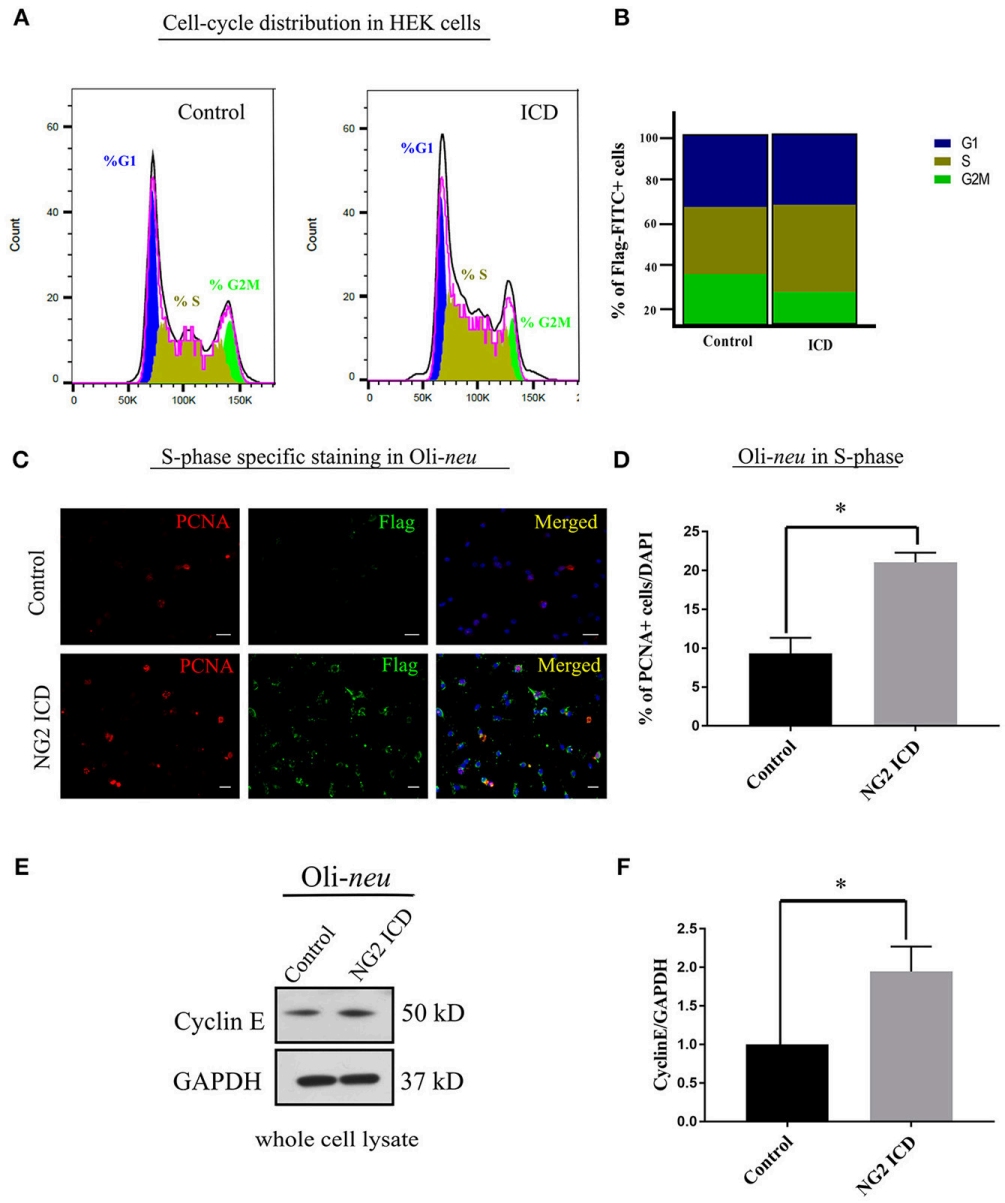
NG2 ICD accumulates cells in S-phase. **(A)** Representative images of DNA histogram analysis for cell-cycle distribution in asynchronized HEK cells transfected with ICD-flag (ICD) or BAP-flag (control). Cells were harvested, fixed, immunostained with Flag-FITC antibody and subjected to FACS analysis. Propidium iodide (PI) was used for DNA staining. Only flag-FITC+ cells were analyzed for PI quantification. **(B)** Quantification of FACS data from HEK cells represented in a stacked bar column shows that ICD promotes the cell population in S-phase by ~10%, while G2M phase fraction is reduced (*n* = 3). **(C)** Oli-*neu* cells immunostained with the anti-PCNA antibody (red) indicate increased PCNA-associated nuclear puncta when transfected with ICD (stained with anti-Flag antibody, green). PCNA is an S-phase specific marker and puncta in nucleus represent active replisomes. Cell nuclei were stained with DAPI (blue). **(D)** Quantification of PCNA staining in Oli-*neu* reveals that the proportion of PCNA+ cells out of total cells (total counted DAPI+) is significantly increased in case of ICD overexpression (21.04 ± 1.26%) compared to control (9.33 ± 2.03%). **(E)** Cyclin E is upregulated during G1 to S-phase progression, Cyclin E total protein levels were checked in Control, and NG2 ICD transfected Oli-*neu* cells. **(F)** Whole cell lysates were subjected to WB analysis, where Cyclin E was increased ~2 fold in ICD compared to control. [Data represents mean ±SEM. Statistical analysis was done by two-tailed paired *t*-test after checking the data is normally distributed by Shapiro-Wilk normality test by PRISM (GraphPad). For **(C,D)** Experiments were done in three independent sets (*n* = 3) where 2,000 events counted for each condition in each experiment for **(B)** and a total of ~250 cells were counted for **D**]. Significance was classified as defined in the Materials and Methods part.

Only FITC+ cells were gated for PI (DNA-binding dye) signal measurement. From the cell cycle distribution, in the ICD transfected population, 45.95 ± 6.53% of the cells were in S-phase compared to 35.41 ± 6.7% in S phase in the control population (Figures 5A,B). Due to low transfection efficiency and technical difficulties, FACS experiments could not be performed on Oli-*neu*. However, to address this issue in Oli-*neu*, cells were immunostained for PCNA (Proliferating cell nuclear antigen) which is an S-phase marker (Figure 5C). PCNA-staining revealed that 21.04 ± 1.26% Oli-*neu* cells were PCNA+ (out of total DAPI+ cells) in the ICD-transfected population. In the control population, 9.33 ± 2.01% Oli-*neu* were PCNA+ (Figure 5D), resulting in a 2-fold increase of Oli-*neu* cells in S-phase after ICD overexpression. 10% more Oli-*neu* were in S-phase after ICD expression, similar to increase in HEK cells. Levels of the G1 to S-phase regulator Cyclin E, increased around 2-fold on the protein level in Oli-*neu* total cell lysates, after NG2 ICD overexpression (Figures 5E,F).

**Figure 6:**
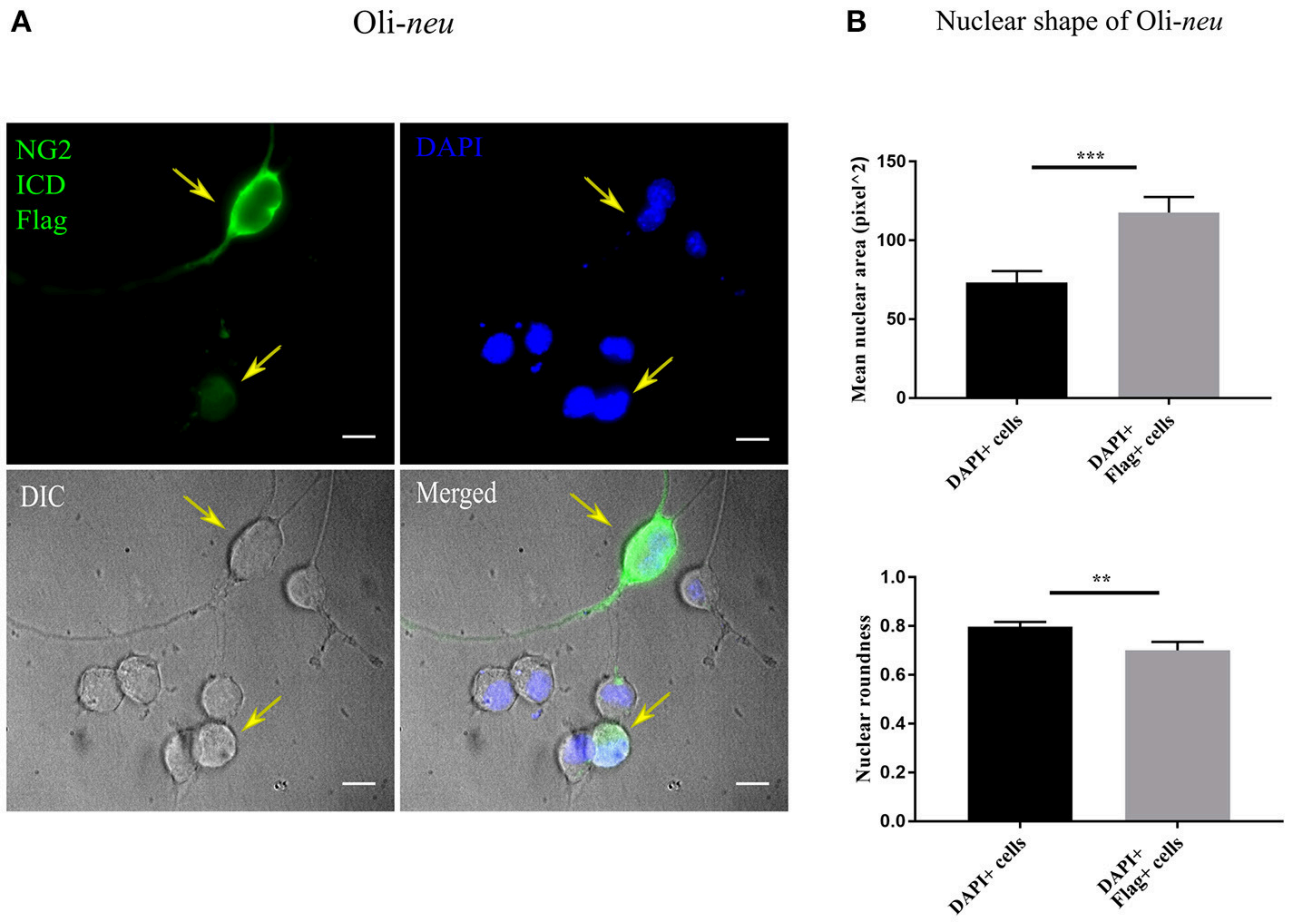
Altered nuclear morphology in ICD-overexpressing cells. **(A)** Immunofluorescent images of Oli-*neu* cells expressing ICD-flag (green), the nucleus was stained with DAPI (blue). The arrows indicate ICD-transfected cells. **(B)** Quantification of nuclear shape based on two indices (ImageJ); nuclear area (unit for area) and roundness (0.0–1.0), revealed that nuclei of ICD-transfected Oli-*neu* were ~1.6 times larger (area) and less round (roundness) than Oli-*neu* from control [Mean values ± SEM was shown in the data. Total 50 cells were counted from three independent experiments (*n* = 3) for each condition. Statistical test was carried out by Mann-Whitney-rank based test]. Significance was classified as defined in the Materials and Methods part.

Recent literature implies that nuclear morphology is correlated with distinct phases of cell-cycle and it is reported that most G1 phase cell nuclei appear round. As cells progress from late S-phase to mitosis, the nuclear shape also changes to a more elongated or dumbbell-shaped appearance (Wang et al., 2016). We examined the nuclear morphology by nuclear area and roundness of cells expressing the ICD. We found a significant difference between mean nuclear area and roundness of ICD transfected cell nuclei compared to controls (Figures 6A,B). While roundness was slightly reduced in ICD overexpressing Oli-*neu* cells, the nuclear area was increased around 1.6 fold in ICD overexpressing cells.

### NG2 ICD increases translation by modulating mTOR signaling components

**Figure 7:**
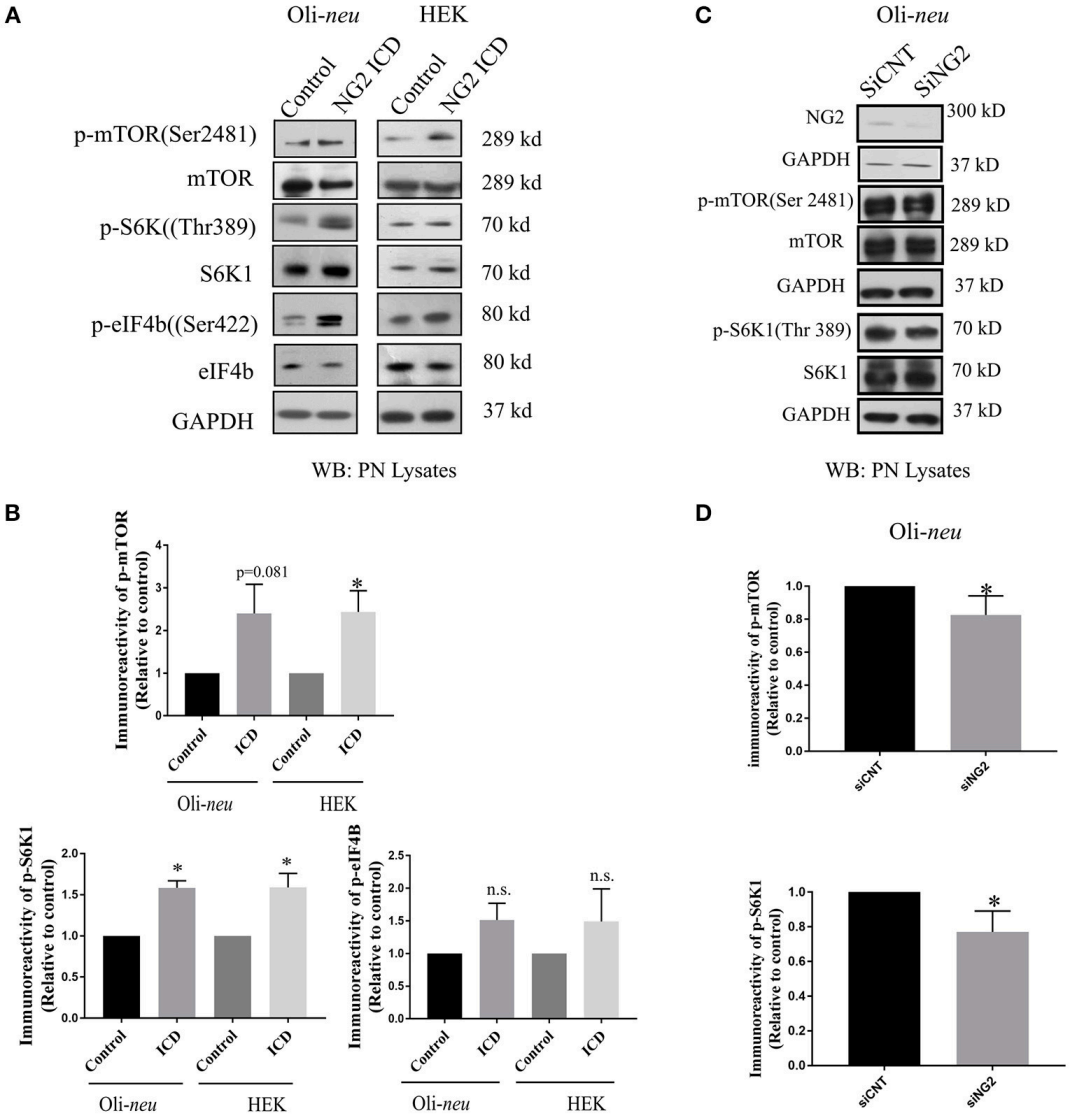
NG2 ICD regulates mTORC1ignaling pathway. **(A,C)** Western blot analysis of post-nuclear (PN) lysate from NG2 ICD transfected HEK (*n* = 3) and Oli-*neu* (*n* = 4) cells compared to empty vector (control). PN lysates were prepared 48 h after transfection. NG2 knockdown was carried out by siRNA transfection in Oli-*neu* cells. **(B)** Quantification of western blot analysis was done by normalizing both phospho-protein and total individual protein against GAPDH (loading control), then individual ratios of phosphorylated over total proteins levels were calculated. Several components of mTOR signaling cascade were regulated by NG2 ICD overexpression. Phospho-specific expression of S6K1 (Thr389) is significantly upregulated in both cell lines and correlates with the increase of phospho-mTOR (Ser2481) levels. The downstream target of pS6K1, p-eIF4B was also increased in both cell lines. **(D)** In the NG2-knockdown experiment, the key components of mTORC1 signaling cascades were checked, and p-mTOR and p-S6K1 both were reduced (~20–25%) in NG2 knockdown (efficacy 80%) samples compared to control. [Data represents mean ± SEM. Statistical analysis was done by two-tailed paired *t*-test after checking the data is normally distributed by Shapiro-Wilk normality test by PRISM (GraphPad)]. Significance was classified as defined in the Materials and Methods part.

We further investigated molecular mechanisms behind the translational and cell-cycle effects mediated by the NG2 ICD. To address this, we studied the expression of the global translation and proliferation regulator mTOR and components of the related signal-cascade. As described before, HEK and Oli-*neu* cells were transiently transfected with ICD or mock vector, and after 48 h, cell lysates were prepared and run on an SDS-PAGE gradient gel followed by WB analysis. In ICD-overexpressing cells, levels of phospho-mTOR (Ser2481) were increased and levels of the phosphorylated form (Thr 389) of the downstream target of mTOR, p70S6K1 were also increased, suggesting that this signaling pathway is a target of NG2 ICD (Figures 7A,B). Active (phosphorylated) S6K1 phosphorylates and inactivates eEF2K resulting in an upregulation of eEF2 protein, and it also phosphorylates and activates eIF4B. In support of this, we found an increase of phospho-eIF4B (Ser422) in ICD-transfected cells (Figures 7A,B). Knock-down of total NG2 protein in Oli-*neu* by siRNA (siNG2) resulted in a decrease of mTOR and p70S6K1 phosphorylation of around 20–25% compared to control (siCNT) (Figures 7C,D).

**Figure 8:**
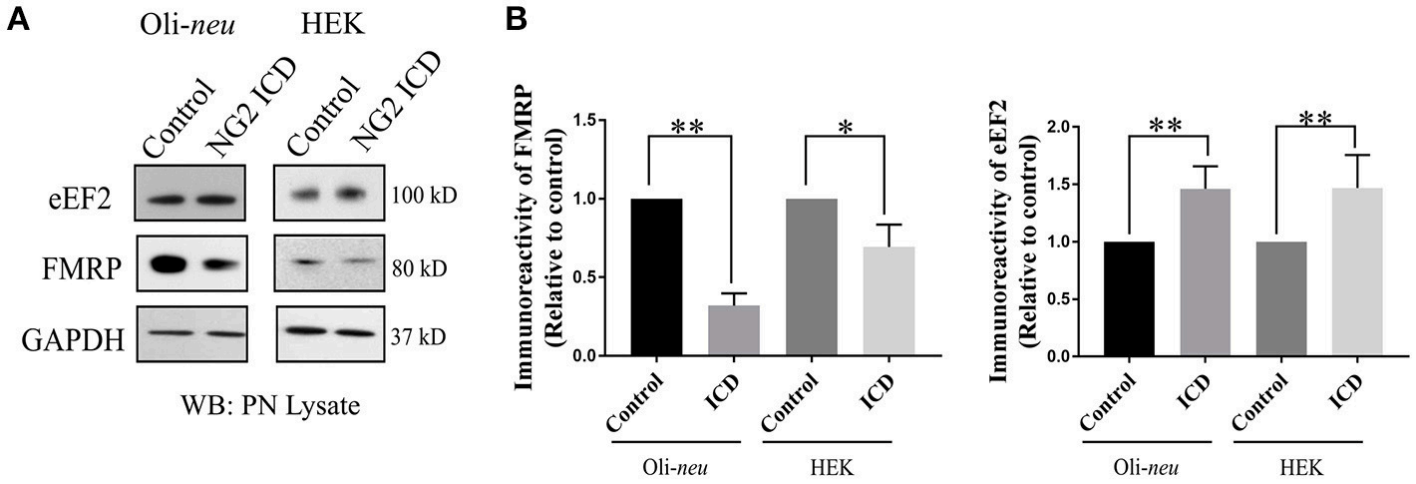
NG2 ICD regulates expression of eEF2 and FMRP. **(A)** Western blot analysis of post-nuclear (PN) lysate from NG2 ICD transfected HEK (*n* = 6) and Oli-*neu* (*n* = 7) cells compared to empty vector (control). PN lysates were prepared 48 h after transfection. **(B)** Total protein levels (normalized against GAPDH) of eEF2 were increased in both cell-lines, while FMRP protein levels were significantly reduced after ICD-overexpression only in Oli-*neu*, both are key regulators of transcription. [Data represents mean ± SEM. Statistical analysis was done by two-tailed paired *t*-test after checking the data is normally distributed by Shapiro-Wilk normality test by PRISM (GraphPad)]. Significance was classified as defined in the Materials and Methods part.

In the nervous system, S6K1 has been reported to phosphorylate FMRP, a protein acting as a general repressor of translation (Narayanan et al., 2008). Mutation of FMRP is the causal link to the intellectual disorder; Fragile-X Mental Syndrome (Santos et al., 2014). FMRP is an RNA binding protein with a wide range of substrates including dendritic mRNAs (Nalavadi et al., 2012) thus FMRP plays a key role in maintaining local translation and neuronal development (Zalfa et al., 2003; Nalavadi et al., 2012; Pasciuto and Bagni, 2014b). In recent studies, Eef2 mRNA has been recognized as an additional substrate of FMRP (Pasciuto and Bagni, 2014a; Richter et al., 2015) and FMRP/Eef2 signaling has been suggested to regulate translation of mRNAs involved in LTD in neurons (Park et al., 2008). Furthermore, FMRP-KO mice show exaggerated protein synthesis (Darnell et al., 2011) as a result of hyperactive S6K1 signaling (Bhattacharya et al., 2012). Intrigued by this reported molecular interplay, we checked the level of FMRP and found significant downregulation (~70%) in ICD-overexpressing Oli-*neu* cells (Figures 8A,B). Downregulation of FMRP was much lower in HEK (~30%) compared to Oli-*neu*. Moreover, the total cellular levels of eEF2 were increased in both NG2 ICD-transfected (Figures 8A,B) cell-types.

## Discussion

### NLS-dependent localization of the NG2 ICD

An initial study by our group investigated the localization of the NG2 ICD. After transfection, most of the protein was localized in the cytoplasm, but lower nuclear levels were also reported (Sakry et al., 2015); in the present study, we confirm this localization pattern. However, immunofluorescent staining of cells expressing an NLS-truncated ICD construct showed only cytoplasmic and no nuclear localization, suggesting an NLS-dependent transport of the NG2 ICD into the cell nucleus. In contrast to the NOTCH ICD which contains a DNA binding domain, the much smaller NG2 ICD does not contain such conserved DNA/TF binding domains implying that a direct function of the NG2 ICD as a transcription factor is unlikely. According to ELM motif database, the NG2 intracellular sequence harbors a predicted site for MAPK interaction, suggesting additional involvement in cell signaling pathways. So far, no nuclear function or binding partner has been identified for the released NG2 ICD, but the predicted intracellular WW4 binding sequence of NG2 (Figure 1C) could be of interest, since WW4 domain-containing proteins have distinct roles, including nuclear functions (Sudol et al., 2001). In this study, we did not use the NLS deletion construct for further experiments because of its low detectable protein levels, reflecting a much lower expression or a much higher degradation rate compared to the NG2 ICD expression construct.

### NG2 ICD increases translation and DNA replication

Based on the results from the ontology analysis of our proteomics data, we investigated the effect of the NG2 ICD on mRNA translation and cell-cycle kinetics.

Strikingly, total translation rate was increased by at least ~70% after NG2 ICD overexpression in Oli-*neu* and HEK cells, but not increased in the presence of an inhibitor of mTORC1. These results demonstrate an essential role of mTORC1 signaling in the increase in translation due to overexpression of the NG2 ICD. Additionally, NG2 DEL expression only slightly increased translation (~22%) in Oli-*neu*. In our present and previous studies (Sakry et al., 2014, 2015) we show that the NG2 CTF but not the ICD, is the major fragment generated after NG2 DEL expression, resulting in much lower levels of overexpressed NG2 ICD protein after NG2 DEL transfection compared to expression of the NG2 ICD construct. The much lower translational increase observed with NG2 DEL compared to NG2 ICD (22 vs. 70%), suggests a protein-level dependent effect of the released NG2 ICD on mTORC1-dependent translational regulation. In support of this, we found that the reduction of total endogenous NG2 protein levels in primary OPCs by siRNA knock-down decreased total translation rate.

Cell cycle analysis demonstrated a shift toward S-phase of cells expressing the NG2 ICD, where the percentage of the cell-population in S-phase increased by about 10% both for Oli-*neu* and HEK cells, reflecting increased DNA replication after NG2 ICD overexpression. We also detected changes in nuclear morphology of ICD-transfected Oli-*neu* concomitant with the observed alterations in nuclear shape reported in S-phase cells (Wang et al., 2016). In the case of Oli-*neu*, the cell number in S-phase increased by 2-fold, interestingly we found that Cyclin E, the major Cyclin upregulated during G1 to S-phase shift (Sgambato et al., 2003; Loeb et al., 2005), is increased around 2-fold on the protein level in Oli-*neu* under the same experimental conditions.

To date, NG2 protein or cleavage fragments have not been reported to modulate translation. However, NG2 expression is characteristic for proliferating cells, both in normal tissue and tumors. OPCs are the only proliferating cell population in the adult unlesioned mammalian CNS, apart from stem cells (Simon et al., 2011; Dimou and Götz, 2014). The ICD-mediated drive toward S-phase progression in OPCs that we report is striking as it has been recently published that stimulation of neuronal circuits causes an increase in adjacent neuronal progenitor proliferation, including NG2+ OPCs (Gibson et al., 2014). Since neuronal activity stimulates NG2 cleavage leading to increased release of the NG2 ICD in the cell cytoplasm (Sakry et al., 2014), our observation of ICD stimulation of cell-cycle progression is of interest in this context.

### NG2 ICD regulates mTOR and FMRP signaling

Since mRNA translation and cell-cycle kinetics were the major biological processes altered upon ICD overexpression, we investigated the potential underlying molecular mechanism behind these regulated biological processes. We started by analyzing the mTOR signaling cascade, as the mTOR pathway is a central regulator of cell growth, proliferation, and survival and growing evidence also supports its role in cell-cycle progression and tumorigenesis (Fingar et al., 2003; Ohanna et al., 2005; Laplante and Sabatini, 2012).

We found an increase of phosphorylated (p-ser2481, active) mTOR in ICD-overexpressing Oli-*neu* and HEK cells, indicating activation by PI3K, which is responsible for phosphorylation of this specific residue (Soliman et al., 2010). Active mTOR, part of the mTOR complex1 (mTORC1), is known to phosphorylate the downstream target S6K1 at the Thr389 residue (Saitoh et al., 2002). Phosphorylated (active) S6K1 phosphorylates several downstream targets including the translation regulators eIF4B and eEF2K (Wang et al., 2001; Holz et al., 2005). Phosphorylated eIf4B promotes initiation of cap-dependent translation (Dennis et al., 2012), while phosphorylated eEF2K (the inactive form) leads to an increased level of the eEF2 protein resulting in elevated translation rates (Wang et al., 2001). Focusing on mTOR downstream targets, we found significantly increased levels of phosphorylated S6K1 in both cell lines after NG2 ICD overexpression. Additionally, total amounts of eEF2 protein and levels of p-eIF4B (the active form) were upregulated in both cell-lines, supporting the activation of the mTOR signaling cascade. Knock-down of endogenous NG2 in Oli-*neu* lead to decreased levels of phosphorylated mTOR/S6K1.

In addition to acting on the translation factors as described above, active S6K1 has been reported to phosphorylate FMRP, a protein highly expressed in the brain (Narayanan et al., 2008). Furthermore, eEF2 is a reported target of FMRP (Darnell and Klann, 2013). In FMRP knockout mice, total eEF2 protein and levels of p-eIF4B were higher than in wild-type mice (Bhattacharya et al., 2012). In line with this observation, we found significant downregulation of FMRP protein levels of 70% compared to the normal protein levels in NG2 ICD-overexpressing Oli-*neu*, but in HEK cells overexpressing the NG2 ICD, FMRP protein levels were only 30% lower. These NG2 ICD-mediated effects on FMRP were thus much stronger in Oli-*neu*, in contrast to the ICD mediated effects on the mTOR and S6K1 pathway which were similar in both cell types.

Fragile X protein (FMRP) is an RNA binding protein with demonstrated roles in mRNA transport, localization, stability, and translation. Mutations in FMRP are a major cause of the human cognitive disorder Fragile-X syndrome. In FMRP knock-out mice, increased S6K1 signaling was reported (Bhattacharya et al., 2012) leading to exaggerated protein synthesis, similar to observations in FXS. Reduced expression of functional FMRP in both FXS patients (Qin et al., 2013) and animal models (Weiler et al., 2004; Gross et al., 2010) leads to excessive protein synthesis in neurons, suggesting that FMRP acts as a general repressor of translation. Since we found reduced FMRP levels and increased translation after NG2 ICD overexpression in Oli-*neu*, it is likely that FMRP also acts as a translational repressor in OPCs, as well as in neurons.

**Figure 9:**
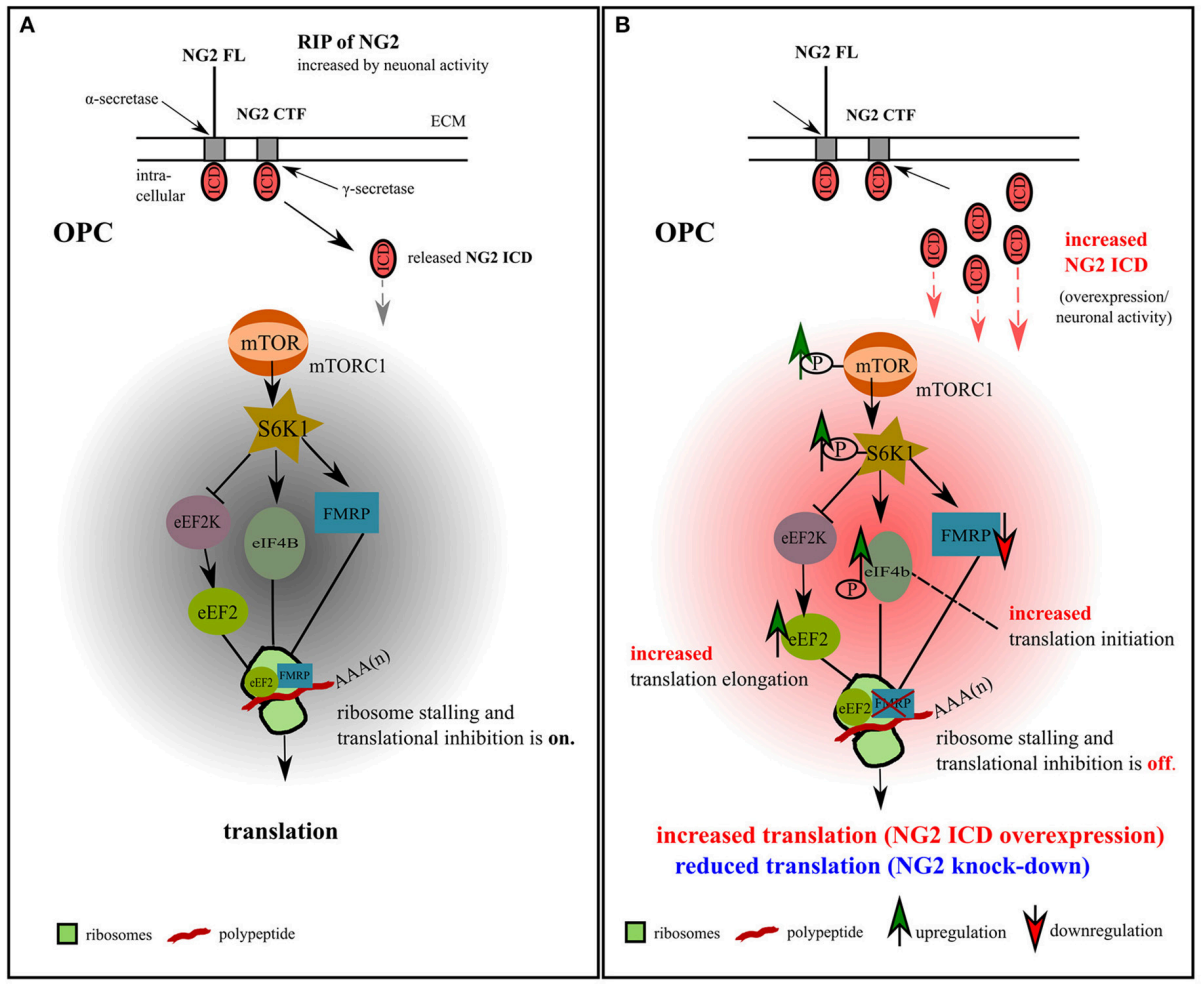
Model for NG2 ICD-mediated intracellular signaling in OPCs. **(A)** We propose the signaling pathway shown for OPCs, based on our previous NG2 cleavage study (Sakry et al., 2014; Sakry and Trotter, 2016) and the present NG2 ICD overexpression and NG2 knock-down study (highlighted in part **B**). The mTOR pathway shown has been suggested by Bhattacharya et al. (2012) for neurons (without NG2 signaling). In OPCs, NG2 full-length (FL) protein undergoes regulated intramembrane proteolysis (RIP) leading to the release of an ectodomain into the extracellular matrix (ECM) and generation of a c-terminal fragment (CTF) by α-secretase activity. γ-secretase activity on the NG2 CTF releases the NG2 intracellular domain (ICD) into the cytoplasm. This NG2 cleavage occurs constitutively in OPCs and can be increased by neuronal activity (Sakry et al., 2014). Under normal conditions, FMRP, which is a translation repressor and target of the mTOR-S6K1 signaling cascade, binds to eEF2 on ribosomal subunits and stalls translation, as suggested for neurons (Bhattacharya et al., 2012). **(B)** Increased NG2 cleavage generates an increased amount of NG2 ICD by γ-secretase activity. NG2 ICD overexpression mimics an increase in products of intracellular NG2 cleavage, elevating levels of phosphorylated (active) p-mTOR/p-S6K1/p-eIF4B, all known downstream components of the mTORC1 signal cascade, and thus indicating an increase in p-eIF4B dependent translation initiation. P-S6K1 phosphorylates and inactivates eEF2K leading to increased levels of active eEF2 protein favoring translation elongation. Increasing levels of NG2 ICD resulted in a higher levels of eEF2 and a reduction of FMRP protein, leading to a reduction of stalled FMRP-eEF2 complexes and relieving translation inhibition. These combined effects on the signaling cascade favor the observed mTORC1-dependent increase of overall translation in OPCs (Figures 4A,B) after ICD overexpression. A reduction of NG2 protein level in primary OPCs leads to a reduction of the total translation rate (Figures 4C,D).

In our study of OPC, we identified two translation-related pathways affected by the NG2 ICD. One is responsible for general translation regulation in cells (mTORC1/S6K1 signaling cascade), and the other is important for regulating local translation and synaptic strength including LTD (FMRP-eEF2) in neurons (Park et al., 2008). Recent studies have shown that FMRP also activates the mTOR-signaling pathway in neurons (Sharma et al., 2010), combining these two signaling cascades. We thus suggest a new signaling paradigm for the NG2 protein in OPCs involving the neuronal network, as summarized in the graphical illustration in Figure 9.

### NG2 ICD signaling in OPCs

Our findings establish a model for NG2 ICD dependent modulation of FMRP/mTOR signaling cascades in OPCs with associated increased translation rate and an increased fraction of the cell population in the S-phase. OPCs are the only glial cell-type that receives direct synaptic input from neurons in all major brain areas (Bergles et al., 2000; Jabs et al., 2005; Mangin and Gallo, 2011; Sakry et al., 2011). Furthermore, cleavage of NG2 leading to the cytoplasmic release of NG2 ICD has been shown to be increased by neuronal activity (Sakry et al., 2014).

The mTOR pathway has been shown to be crucial for OPC differentiation to myelinating oligodendrocytes (Tyler et al., 2009; Zou et al., 2014). However, NG2 is downregulated during early OPC differentiation (Nishiyama et al., 2009; De Biase et al., 2010; Kukley et al., 2010) making it unlikely that regulation of mTOR by NG2 is contributing to OPC differentiation.

### NG2 ICD signaling in tumors

Interestingly, dysregulation of the FMRP/Akt/mTOR pathway has been reported to promote tumorigenesis (Rajasekhar et al., 2003; Lucá et al., 2013) and impaired mTOR cascade has especially been linked to Glioblastoma, the most common form of primary brain tumor (Akhavan et al., 2010; Li et al., 2016). NG2 is abundantly expressed by highly proliferative tumor cells in melanomas and gliomas (Chekenya et al., 2008; Al-Mayhani et al., 2011). The migration-promoting function of NG2 (Binamé et al., 2013) as well as the binding to OMI/HtrA2 (Maus et al., 2015) are features favoring NG2 expression by tumor cells promoting invasion and increasing resistance to oxidative stress. NG2 is included in a pool of several antigens used in a vaccine therapy against glioblastoma multiform, which reduces tumor growth (Poli et al., 2013). Additionally, OPCs are discussed as the major cells of origin for gliomas (Liu and Zong, 2012). NG2 is also involved in regulating symmetric vs. asymmetric cell-division of OPCs, the mode of division influences the likelihood of an OPC to become a tumor cell (Sugiarto et al., 2011).

Our findings of increased translation and DNA-synthesis (S-phase) by NG2 ICD correlates with the high proliferation and expression rates of these NG2-expressing tumors and is therefore likely to be one contributing factor to increased translation and DNA-synthesis within these cancers. One could speculate, that targeting NG2 as an antigen in the vaccine therapy described above may affect NG2 signaling and modulate the signaling-pathway presented here. It could further explain part of the reduction in tumor growth observed during such therapy by reduced translation and proliferation effected by NG2 signaling.

## Summary

In summary, our results add new insights to the role of NG2 cleavage in OPC and tumor signaling pathways. In addition to effects of the released NG2 ectodomain on the glutamatergic properties of neurons (Sakry et al., 2014), we show here that the released NG2 ICD can signal as a functional domain affecting translation and cell-cycle kinetics. These effects are important both for OPC under normal physiological conditions as well as NG2-expressing tumors. Our results imply translational regulation of localized (synaptic) mRNAs by FMRP in OPCs that can be influenced by the neuronal network.

## Author contributions

TN and DS conceived and designed the experiments. TN performed the experiments and analyzed the data. DS and JT supervised the experiments. TN, DS, and JT wrote the manuscript.

### Conflict of interest statement

The authors declare that the research was conducted in the absence of any commercial or financial relationships that could be construed as a potential conflict of interest.
